# Exercise Stress Echocardiography: A Dynamic Assessment for an Evolving Landscape

**DOI:** 10.31083/RCM47079

**Published:** 2026-01-16

**Authors:** Eduardo M. Vilela, Francisco Sampaio, José Ribeiro, Ricardo Fontes-Carvalho

**Affiliations:** ^1^Cardiology Department, Unidade Local de Saúde de Gaia e Espinho, 4434-502 Vila Nova de Gaia, Portugal; ^2^Faculdade de Medicina, Universidade do Porto, 4200-319 Porto, Portugal; ^3^RISE-Health, Departamento de Cirurgia e Fisiologia, Faculdade de Medicina, Universidade do Porto, 4200-319 Porto, Portugal

**Keywords:** cardiology, echocardiography, echocardiography stress, exercise

Since the first reports on the clinical use of echocardiography, this technique (versatile and without ionizing radiation exposure) has undergone major developments, becoming a fundamental diagnostic examination across various areas of medicine [1, 2, 3, 4, 5]. Simultaneously, the use of physical exercise to provide a dynamic assessment of the cardiovascular system has also received increasing interest [2]. These fields have become progressively intertwined with the development of exercise stress echocardiography (ESE), which has broadly expanded since its first descriptions to provide a comprehensive assessment of cardiovascular physiology [3, 4, 5, 6]. While other stress methodologies (including dobutamine, vasodilators such as adenosine and dipyridamole, or pacing) have since emerged, thus enhancing the adaptability of this test, by providing a physiological stressor exercise can allow a unique view of several pivotal phenomena [4, 5, 7]. This is underscored by its depiction as a preferred modality in those able to exercise, both in ischemic heart disease (IHD) but also in other applications [4, 5, 7]. Although IHD and the analysis of regional wall motion abnormalities (RWMA) have classically been a focal point of ESE, data has progressively shown its importance across other clinical entities (Fig. 1) [4, 5, 6, 7, 8, 9].

**Fig. 1.**
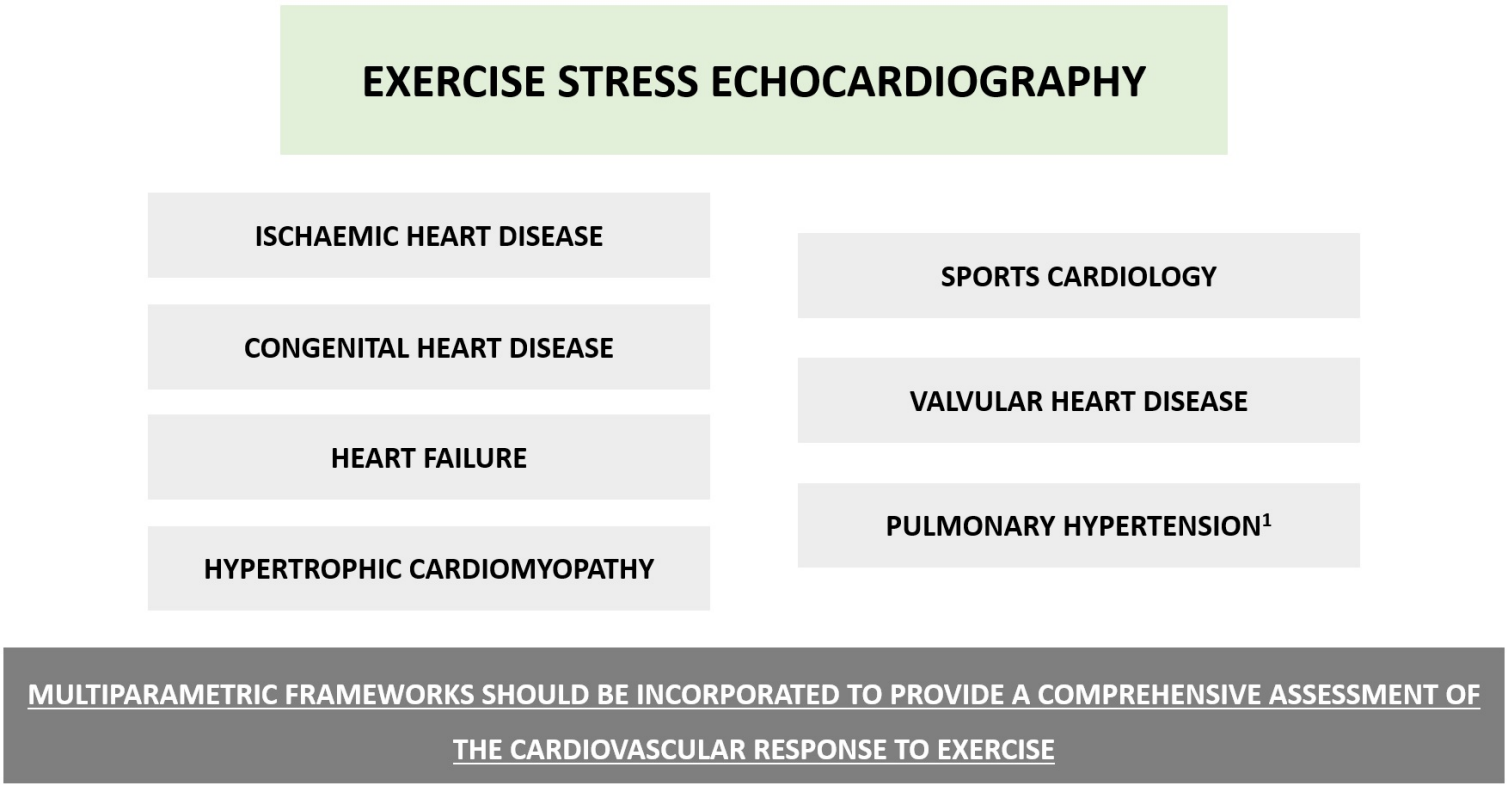
**Overview of some of the main applications of exercise stress echocardiography**. Beyond ischemic heart disease, numerous other potential applications have been described for exercise stress echocardiography. An overview is depicted (non-exhaustive list), whereas other ancillary uses such as Kawasaki disease or the assessment of left atrial dynamics have also been described. Although an analysis of regional wall motion abnormalities remains a core part of this test, incorporating data from different components such as functional capacity, blood pressure or electrocardiographic analysis (namely arrhythmias and heart rate response), as well as lung water and other echocardiographic-derived parameters (see main text for details) can assist in providing an integrated assessment of the cardiovascular response. Pulmonary hypertension^1^, particularly in those with systemic sclerosis.

In hypertrophic cardiomyopathy (HCM), ESE can be useful to assess left ventricular outflow tract obstruction (LVOTO) as well as valvular abnormalities [7]. While resting and provocative echocardiography is the first-line technique when assessing LVOTO, European and American guidelines recommend (class I, level B) ESE in symptomatic individuals with a gradient <50 mmHg to assess dynamic variations [7, 9]. During exercise, mitral regurgitation may also be assessed, and in select cases, stress echocardiography (SE) may allow assessment of myocardial ischemia [7]. American guidelines also suggest (class 2a, level C) performing ESE in asymptomatic patients with gradients <50 mmHg to assess dynamic changes, which may impact management such as the use of concomitant medications or the patients’ hydration status [9]. The use of dobutamine alone may lead to intracavitary gradients [10]. This should be noted because contrary to exercise, dobutamine stress should not be employed in this setting as it is non-physiological (and could lead to gradients in normal subjects), and it may be poorly tolerated [7, 9, 11].

In heart failure (HF), SE can be of interest to assess ischemia (class 2b, level B in European and American guidelines, alongside modalities such as magnetic resonance imaging or nuclear tests) and also as a diagnostic tool [12, 13, 14]. This can be helpful when addressing unexplained exertional dyspnea in the face of normal resting data, since HF with preserved ejection fraction (HFPEF) patients may have normal resting left ventricular (LV) filling pressures, but abnormal responses during exercise [14, 15]. While different stressors may be suitable for ischemic assessment, exercise is particularly useful when considering a diagnosis of HFPEF, though challenges in assessing diastolic function should be considered [14]. Inability to exercise, difficulties in E/e’ analysis (due to fusion during tachycardia) or in the assessment of tricuspid regurgitation (TR) (particularly at higher workloads) should be considered [6, 14]. Guidelines from the American Society of Echocardiography discourage the use of dobutamine when performing a dynamic assessment of diastolic function [15].

In valvular heart disease, ESE may be useful for diagnostic purposes in patients with unexplained dyspnea, and in asymptomatic individuals or those with discrepancies between symptoms and resting findings [16]. In patients with aortic stenosis and mitral regurgitation (where prognostic roles have been especially studied) and also in mitral stenosis, it can be applied in selected settings to enhance risk stratification [16].

Sports cardiology is another area where ESE may provide important insights [17]. This can provide a functional evaluation in selected individuals, such as those with borderline or uninterpretable prior exercise test results [17]. Other areas of interest include competitive athletes with an anomalous aortic origin of a coronary artery or symptomatic individuals with myocardial bridging [18]. In these patients, it should be noted that vasodilator stress testing is not recommended [18]. ESE can also be used for the differential diagnosis between exercise-induced cardiac remodelling (a feature of the athlete’s heart) and pathological settings (such as dilated cardiomyopathy) [17, 19, 20, 21]. Parameters such as increases in LV ejection fraction during exercise and electrocardiographic findings such as the development of exercise-induced arrhythmias, should be coupled with other data to provide an integrated assessment in this oftentimes challenging scenario [17, 19, 20, 21]. Finally, entities such as congenital heart disease (including the assessment of right and LV function, or TR) or pulmonary hypertension (namely in those with systemic sclerosis, to assist in decisions concerning right heart catheterization) should also be considered [4, 5, 6, 22].

As previously noted, analysing RWMA (throughout the exam, with a scoring system) has been a cornerstone of SE, while ancillary data such as that derived from ejection fraction or ventricular dimensions also provides data that can be used for risk stratification [4, 5, 6, 23]. Perfusion imaging with ultrasound enhancing agents has emerged as a potentially useful tool when assessing IHD, as described in current guidelines, though some limitations should be acknowledged [4, 5, 8, 23, 24]. Other parameters have also been recognized as instrumental for an integrated assessment, including exercise capacity (a powerful predictor of events), chronotropic response, and exercise hemodynamics [4, 5, 17, 25, 26]. Combining data on LVOT obstruction with blood pressure and arrhythmias during exercise is pivotal when stratifying risk in diseases such as HCM [17, 27]. Data on lung water and pulmonary congestion (with a focus on the presence and density of B-lines), LV contractile reserve and coronary flow velocity reserve have also been described in frameworks such as the ABCDE protocol [5, 6, 23]. This can be further expanded by including additional parameters, illustrating the global nature of this technique [5, 23]. While exercise is a first-line choice, specific settings may trigger different stressor selections when addressing distinct components [4, 5].

Novel challenges and innovations continue to advance ESE [4, 5, 23, 28]. While appropriate protocol selection, image quality, electrocardiographic findings and standardization have been previously discussed, the incorporation of technologies such as artificial intelligence (aiming to improve accuracy and reproducibility) and the increasingly recognized role of environmental burden or cost considerations continue to shape paradigms for this highly useful test in contemporary clinical practice [4, 5, 23, 28, 29, 30]. Furthermore, challenges such as the validation and application of novel technologies, or individualization based on large-scale outcome studies across distinct populations are some of the unmet needs in this field [4, 5, 23, 29]. In a landscape where personalized decision-making based on a tailored assessment of the individual patient is paramount, ESE is set to continue to evolve as a central tool in cardiovascular medicine.

## Abbreviations

ESE, exercise stress echocardiography; HCM, hypertrophic cardiomyopathy; HF, heart failure; HFPEF, heart failure with preserved ejection fraction; IHD, ischaemic heart disease; LV, left ventricular; LVOTO, left ventricular outflow tract obstruction; RWMA, regional wall motion abnormalities; SE, stress echocardiography; TR, tricuspid regurgitation.
